# The Contribution of Elastic Wave NDT to the Characterization of Modern Cementitious Media

**DOI:** 10.3390/s20102959

**Published:** 2020-05-23

**Authors:** Gerlinde Lefever, Didier Snoeck, Nele De Belie, Sandra Van Vlierberghe, Danny Van Hemelrijck, Dimitrios G. Aggelis

**Affiliations:** 1 Department Mechanics of Materials and Constructions, Vrije Universiteit Brussel (VUB), Pleinlaan 2, 1050 Brussels, Belgium; didier.snoeck@ugent.be (D.S.); Danny.Van.Hemelrijck@vub.be (D.V.H.); Dimitrios.Aggelis@vub.be (D.G.A.); 2 Magnel-Vandepitte Laboratory for Structural Engineering and Building Materials, Department of Structural Engineering and Building Materials, Faculty of Engineering and Architecture, Ghent University, Tech Lane Ghent Science Park, Technologiepark Zwijnaarde 60, 9052 Ghent, Belgium; nele.debelie@ugent.be; 3 Polymer Chemistry & Biomaterials Research Group, Centre of Macromolecular Chemistry, Ghent University, Krijgslaan 281 S4-Bis, 9000 Ghent, Belgium; sandra.vanvlierberghe@ugent.be

**Keywords:** acoustic emission, ultrasound, hydrogel, nanosilica

## Abstract

To mitigate autogenous shrinkage in cementitious materials and simultaneously preserve the material’s mechanical performance, superabsorbent polymers and nanosilica are included in the mixture design. The use of the specific additives influences both the hydration process and the hardened microstructure, while autogenous healing of cracks can be stimulated. These three stages are monitored by means of non-destructive testing, showing the sensitivity of elastic waves to the occurring phenomena. Whereas the action of the superabsorbent polymers was evidenced by acoustic emission, the use of ultrasound revealed the differences in the developed microstructure and the self-healing of cracks by a comparison with more commonly performed mechanical tests. The ability of NDT to determine these various features renders it a promising measuring method for future characterization of innovative cementitious materials.

## 1. Introduction

Recently, there have been many developments in cementitious media, especially in the field of admixtures aiming to enhance mechanical properties, but mostly to extend durability and in doing so, to improve sustainability. In the process of developing innovative materials, monitoring techniques play an important role. Specifically, elastic wave methods allow non-invasive and non-destructive characterization of the mechanical properties (i.e., direct calculation of stiffness and correlation with the strength) as well as the monitoring of processes like setting and hydration of concrete with admixtures by means of active (ultrasound) or passive elastic waves (AE).

In the present paper, cementitious materials with different admixtures are tested. These admixtures are superabsorbent polymers (SAPs), nanosilica (NS) and a combination of both. SAPs are applied in concrete mainly to prevent shrinkage cracking by internal curing [1,2,3,4,5]. They function by initially absorbing (extra) water and releasing it to the cementitious matrix at a later stage, when the evaporation rate as well as the chemical hydration reaction reduce the amount of available water in the mixture and increase the capillary pressure in the system. In a normal situation, when the pressure becomes too high, air enters into the system, demonstrated by a sudden drop of capillary pressure and signifying a high risk for shrinkage cracking [6]. This is also escorted by AE bursts recorded within the same time frame of the pressure drop [7]. In case SAPs are present, the absorbed water inside the SAPs is released due to the increase of capillary pressure. This smoothens the effect of evaporation rate, ideally avoids the drop in internal relative humidity, and allows for continued hydration, enabling the cementitious material to resist the tensile forces leading to cracking. Practically, SAPs eliminate cracking as was revealed from the dedicated restrained ring tests in the time frame of the study (1 month) [8]. The contribution of the SAPs in controlling the shrinkage cracking is undeniable. However, they impose a certain reduction in mechanical properties due to the increase of the porosity. The dry SAP grains under study are normally 100 ± 21 μm in size while after water absorption, their size can reach up to 257 ± 55 μm, as they absorb approximately 26 times their mass in water when included in a cementitious mix. This value was obtained by measuring the flow of fresh mortars with and without SAPs using the flow table test [9]. The amount of additional water, necessary to obtain an identical flow of SAP mortars compared to the reference material, determines the absorption capacity of the SAP in the studied environment. After the water is drained back to the cementitious matrix, cracking is avoided [10] but the microstructure is affected by the remaining cavities which are a permanent part of the hardened microstructure. Recent results have shown a decrease of the order of 20% in compressive strength and flexural strength for mixtures with SAPs compared to the reference mix without SAPs [11,12]. To compensate for the reduction in strength, nanosilica (NS) particles are used in the mixtures. NS has shown the ability to increase the strength of a cementitious material due to its large surface area, which provides nucleation sites for the hydration of cement, early pozzolanic reaction and filler action. Recent results show that actually NS particles help to restore the mechanical properties in mixes with SAPs to the level of the reference material, while at the same time, the mixes benefit by the cracking mitigation action of SAPs [8].

SAPs are not only used as an admixture to mitigate autogenous shrinkage [13], they are also interesting materials to obtain sealing and healing characteristics [14,15]. Upon crack formation, the SAPs absorb moisture and/or fluids and this can be provided to the cementitious matrix to stimulate further hydration, pozzolanic activity and calcium carbonate crystallization, up to minimally 8 years of age [16]. The further hydration is promoted by nearly 40% compared to the reference cementitious material [17], and can even be repeated for a second healing cycle [18]. The healing characteristic is an interesting feature and requires high amounts (1 m% of binder) of SAPs to be added, although detrimental for the mechanical properties [19,20], which can be counteracted by the addition of NS [21].

The present paper discusses the non-destructive techniques (NDT) used to monitor the material in three phases. First, the fresh stage is evaluated, where the material is curing with simultaneous monitoring by AE. Secondly, ultrasound is used to check the elastic properties of the media in the hardened stage, as the microstructure is significantly modified. Finally, the self-healing stage is monitored, when the specimens are subjected to wet-dry curing cycles to check the potential for crack closure and restoration of mechanical properties due to the action of SAPs that can maintain water and lead to a second stage of hydration and promote the precipitation of CaCO_3_ inside the crack. For the first time in literature, AE is shown sensitive to the activity of SAPs, allowing to monitor the whole duration of internal curing, which so far was only possible with expensive and cumbersome neutron tomography and nuclear magnetic resonance (NMR) testing [22]. In addition, it is the first time that elastic wave measurements indicate the mechanical healing due to the wet-dry cyclic curing, which is later on confirmed by mechanical reloading. In the following section, a brief introduction of elastic wave NDT for the specific applications is provided.

## 2. Elastic Wave NDT in Cementitious Media

### 2.1. Acoustic Emission in Fresh Concrete

Acoustic emission (AE) monitoring has been applied mainly in the last two decades for monitoring of fresh cement paste. Sources that have been targeted include grain settlement, water mobility, hydration reaction and cracking. Some studies indicated start or peak of AE activity during the calorimetric temperature peak that implies relation to the hydration reaction [23,24,25]. However, other studies [26,27] showed the large majority of recorded hits occurring earlier than this peak, leading to the conclusion that significant processes (possibly of lower intensity and thus more difficult to register) occur from as early as the mixing time, much before the chemical reaction of hydration initiates and any heat is developed. Differences in the acquisition equipment (including sensor frequency range and sensitivity), the coupling (with or without waveguide) and the specimen size do not allow for robust conclusions relatively to the original sources. Recent studies showed that individual physical mechanisms like bubble creation in the fresh cementitious matrix and aggregate impacts can be recorded as AE events [28], while it was verified that most of the AE activity during the first 2 h after mixing originates from cement grains settlement [7]. Due to its sensitivity down to the attoJ (10^−18^ J) level, AE is influenced by the size of the grains (fly-ash suspension with mean grain size of 57 μm induces lower frequencies and higher energies of AE than normal cement with average grain size of 12 μm) during settlement (up to 2–3 h after mixing). Furthermore, AE energy exhibits peaks close to the moment of capillary pressure breakdown, giving a good indication when the risk of plastic cracking increases. Therefore, the reception of high energy AE bursts during this stage indicates the starting of the detrimental action of cracking and allows external curing treatment to mitigate it [7]. A recent review on this topic is composed by Aggelis et al. [29].

### 2.2. Ultrasonic Assessment of Hardened Cementitious Media

Elastic waves have been more widely used for characterization of hardened cementitious media resulting in a vast literature on the subject. Indicatively, apart from the well-known general correlations of pulse velocity to strength [30,31], phase velocity has shown sensitivity to frequency and mix parameters like the water and aggregate content [32]. In addition, the amount of heterogeneity in the form of actual or simulated damage alters the wave characteristics decreasing the wave velocity and amplitude [33,34,35,36]. 

In addition, elastic waves have been used in certain cases to evaluate the repair effectiveness in concrete materials and structures [37,38,39]. The wave velocity and amplitude are restored, while this has also been applied in concrete with a self-healing vascular network, showing restoration of wave parameters for healed cracks of width up to 500 μm [40]. In the following section, the aforementioned elastic wave techniques are used for monitoring of all stages of the materials’ life. Focus is given on the NDT aspect of the study, while preliminary results concerning the material properties have been recently published [21].

From the monitoring point of view, measurements were conducted in three stages:

(1) During the hydration of mortar specimens in order to check the AE activity of the modified and reference mixes and specifically monitor the action of SAPs for the first time in literature;
(2) elastic wave measurements on the sound material after 28 days to check the effect of the microstructure on the elastic properties;
(3) elastic wave measurements during the healing cycles to examine in a simple way if the mechanical properties are restored.

## 3. Experimental Details

### 3.1. Materials and Mechanical Testing

Four mortar mixtures were made: a reference mixture, a mixture holding SAPs, a mixture holding nanosilica and finally a mixture combining both SAPs and NS. The cement used for all mixtures is a high-strength ordinary Portland cement, CEM I 52.5 Strong (Holcim, Nivelles, Belgium). To obtain the reference mortar mixture, cement, river sand and tap water were added in a proportion of 1:2:0.35. To allow for an easier compaction, a superplasticizer was included at an amount of 0.4% by weight of the binder.

The superabsorbent polymer used in this research is a copolymer of acrylamide and sodium acrylate, produced by bulk polymerization. The SAP presents the ideal characteristics for internal curing purpose with a particle size equal to 100 ± 21.5 µm [4]. The swelling capacity of the SAP is equal to 305.0 ± 3.7 g/g SAP in demineralized water and 61.0 ± 1.0 g/g SAP in cement filtrate [22], measured following the RILEM recommendations [41]. The necessary amount of SAPs for efficient mitigation of autogenous shrinkage can be calculated by means of Powers’ hydration model [13] to obtain the highest possible degree of hydration. In case of the reference mixture under study, an amount of 0.24% by mass of the binder should be added together with 26 g of water per gram of SAP, leading to an entrained additional amount (w/c)_e_ of 0.063. However, it was chosen to lower the amount of SAP included to the mortar mixtures to an amount of 0.2% by mass of the binder, to partially mitigate autogenous shrinkage and limit the reduction in mechanical properties. Compared to the reference mixture, the amount of superplasticizer was kept constant in the SAP mixture and the workability was the same in all mixtures (flow value of 138 ± 1 mm).

To counteract the decrease in compressive strength, caused by the formation of macropores after water release from the SAPs, a nano-reinforcement was introduced. The nanomaterial used was a colloidal nanosilica, containing 40% of synthetic amorphous silica in a water solution. The nanosilica particles have a nominal diameter of approximately 12 nm and a specific surface area between 18 and 258 m²/g. Cement was in this case replaced by nanosilica in an amount of 2% by mass of cement, so that a constant mass of binder was maintained. Also, the amount of superplasticizer added was increased to 0.76% with respect to the total weight of the binder material to account for the decrease in flowability of the fresh mortar caused by the nanoparticles. All mixtures showed the same workability. Table 1 summarizes the mixture proportions of the mortar blends used throughout this study.

To obtain the mechanical properties of the various mixtures, three prism specimens measuring 40 mm × 40 mm × 160 mm were cast per mixture and cured in plastic foil at 20 ± 1 °C. Their compressive strength was measured according to ASTM C349-18 [42]. The average densities after 28 days of curing and the compressive strengths are summarized in Table 2, along with the standard deviations. It can be seen from the results that the addition of SAPs indeed has a strong influence on the mechanical performance, decreasing the compressive strength, while the use of NS restores the compressive strength.

### 3.2. Acoustic Emission Monitoring

**Figure 1:**
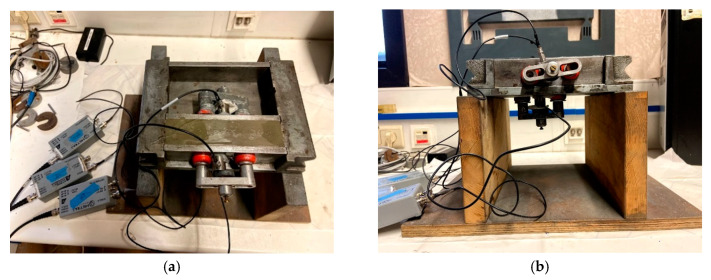
Set-up for acoustic emission monitoring: (**a**) top view showing two sensors with magnetic holders at opposite sides of the beam and (**b**) a side view revealing the bottom sensor.

To monitor the hydration process, a metallic mold equipped with three piezoelectric sensors was used. The sensors were of type R15α and had an operating frequency between 50 and 400 kHz and a resonance frequency at 150 kHz. The three sensors were placed along the sides of a prism specimen of 40 mm × 40 mm × 160 mm: two of them were oppositely attached to the longitudinal faces of the beam mold, while the third was placed on the bottom surface. The fresh mortar specimens were monitored for a period of three days in sealed conditions. The set-up is shown in Figure 1.

### 3.3. Surface Wave Measurements

**Figure 2:**
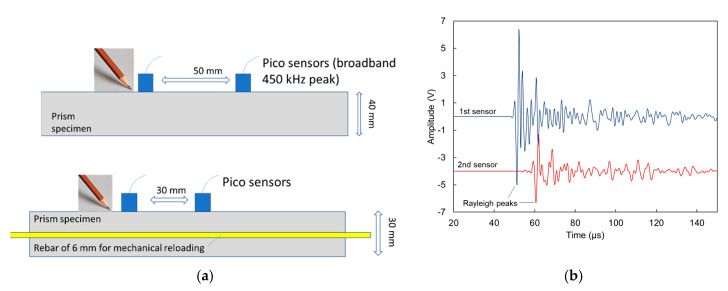
(**a**) Experimental set-up for surface wave measurements; (**b**) shows typical waveforms after pencil lead break in front of the first sensors.

In order to conduct elastic wave measurements, two pico sensors were placed on the top of the specimen. Pico sensors have their sensitivity peak at 450 kHz but they are broadband sensors, which operate between 50 to 800 kHz. The two sensors were located at a distance of either 50 mm or 30 mm, depending on the type of specimen used (plain for sound property determination and with steel rebar for mechanical loading and reloading purposes, respectively), and the excitation took place through a pencil lead break at a distance of approximately 1 cm from the first sensor as shown in Figure 2a. Typical signals received by the two sensors on sound material are depicted in Figure 2b. The signal in the 2nd sensor arrives later and is much lower compared to the 1st, due to the extra distance. Considering the delay between the onset of the two waveforms, the longitudinal wave velocity could be calculated. In addition, by identifying the dominant Rayleigh cycle in both waveforms, the Rayleigh wave velocity was also calculated, as the ratio of the sensor distance over the time delay between the characteristic points (Figure 2b). Apart from the surface measurements, ultrasonic measurements were conducted with a commercial high-power device through the longitudinal axis as well at a resonant frequency of 54 kHz on the specimens without rebar.

The surface wave measurements on sound material were conducted on prism specimens of dimensions 40 mm × 40 mm × 160 mm, six per mixture, after 28 days of curing. After measurement of the sound material, these prisms were cracked by means of a three-point bending test. A carbon fiber reinforced polymer (CFRP) laminate allowed the two halves of the prism specimens to be kept together (at the position of the CFRP laminate). A metal framework was then placed around the specimen, following the procedure described in [43]. By means of restraining with the metal framework, the crack width opening was decreased to approximately 150 µm for all specimens. The determination of the crack width was done by microscopic measurements, using a Leica S8 APO optical microscope equipped with a DFC 295 camera. Along the crack mouth opening, three positions were chosen and a micrograph was taken. In each of these pictures, five measurements of the crack opening were conducted, leading to a total of 15 measurements per specimen.

**Figure 3:**
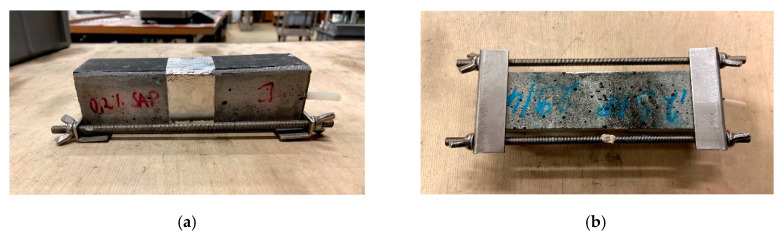
Prism specimens cracked in three-point bending with metal framework for restrained crack opening: (**a**) side view and (**b**) bottom view showing the crack in the center of the specimen.

Afterwards, wet-dry healing cycles were applied on five out of six specimens and this for all mixtures, for a period of 28 days. These cycles consist of 1 h submersion in water at 20 ± 1 °C and 23 h of dry conditions at a relative humidity of 60 ± 5% and temperature of 20 ± 1 °C for a period of 28 days. The remaining specimen was kept in dry conditions, identical to the environmental conditions of the dry period during the healing cycles. Measurements of the crack width were repeated in the exact same 15 locations as described above after 3, 7, 14 and 28 days of wet-dry curing. By means of these microscopic measurements, visual crack closure, implying possible healing of cracks, can be seen. A side and bottom view of the specimens with metal framework can be seen in Figure 3a,b, respectively. A more detailed explanation on the experimental testing procedure and the results can be found in [21].

**Figure 4:**
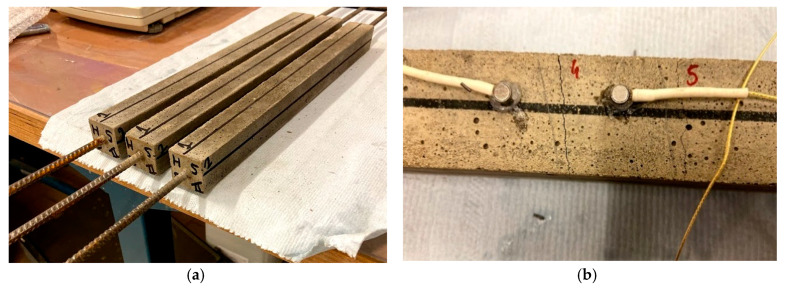
Specimens used during wet-dry healing cycles: (**a**) geometry of the specimens with central rebar and (**b**) positioning of the sensors around the crack opening.

For the further examination of healing by means of mechanical loading and reloading, although the sensors and excitation remained the same, the specimens’ geometry was slightly modified (cross-section of 30 mm × 30 mm, and length of 360 mm) and a thin steel rebar of 6 mm diameter and a length of 700 mm was embedded at casting [44]. Tensile loading and reloading were performed by clamping the reinforcement bar of the test specimens into an Instron 5982 Floor Model Testing System (Instron GmbH, Darmstadt, Germany). The capacity of the load cell is 100 kN and a uniaxial tensile load was applied at a speed of 0.01 mm/s. In the loading stage, the tensile load-displacement response presents initially a linear increase, characterized by the stiffness of the composite beam. When cracking occurs, a sudden drop in the load is noticed. This occurs for every additional crack, until no new cracks were formed and the final part of the load-displacement curve shows the capacity of the steel rebar only. The displacement was increased further on, until a certain opening of the cracks could be maintained after release of the applied load. The opening of the initial cracks was then also measured by means of microscopy and this in five locations on each of the four sides of the mortar specimen, leading to a total of 20 measurements per crack. Upon reloading of a cracked specimen, the response is identical to the final part measured in the loading stage. However, when healing of cracks has taken place, following the same healing procedure as described above, the load-displacement curve could show a regain in stiffness as well as the occurrence of new cracks. Figure 4a shows typical specimens of the latter case, while Figure 4b shows the location of the sensors in either side of a crack after mechanical loading. Three specimens were cast per mixture. The test was performed after 28 days of curing in plastic foil, at a room temperature of 20 ± 1 °C.

During the aforementioned mechanical loading, multiple cracks initiated in the mortar matrix and measured between 50 and 500 µm. At this moment, one or two cracks per specimen were arbitrarily chosen to be followed up during the wet-dry healing cycles. After choosing the cracks to be monitored, the sensors were placed around these chosen cracks, at 30 mm apart. Several surface wave measurements, consisting of a pencil lead break test as explained in Section 3.3, were then conducted and repeated after 3, 7, 14 and 28 days of wet-dry curing, close to the end of the dry period. After this 28-day period, mechanical reloading was performed to investigate whether a regain in mechanical properties could be obtained.

## 4. Results

### 4.1. Acoustic Emission Monitoring During Hydration

**Figure 5:**
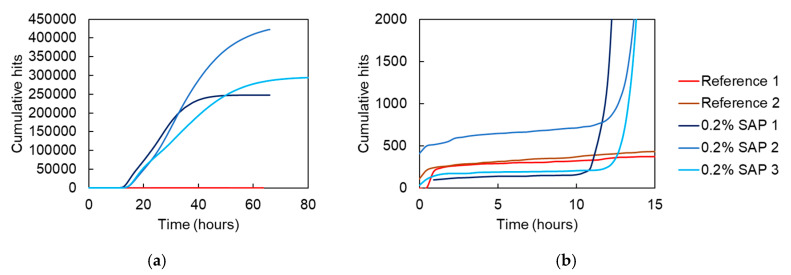
Cumulative hits versus time of various reference (2 replicates) and SAP-containing mortar prisms (3 replicates) in (**a**) and a zoom during the first 15 h in (**b**).

Results of the cumulative AE activity are seen in Figure 5a, where various curves of reference mortar and mortar with SAPs are included for a monitoring period of approximately three days. It is obvious that the SAPs’ modified mixtures exhibit much higher activity that starts to evolve at approximately 11 h after mixing. According to previous studies, this is practically the time when SAPs start to release their water back to the mixture [22]. AE monitors the whole period of SAP contribution, showing that the phenomenon comes to completion after 40 h, again in correlation with literature [22]. The AE activity may come from the water flow in the porosity of cement as well as from the detachment of the SAPs from their cavity as they shrink. While this is still under consideration, it is the first time that AE is used to monitor the phenomenon, which so far could be traced only by cumbersome and expensive neutron tomography and NMR [22]. 

Figure 5b focuses on the first 15 h of AE, where the nearly vertical increase due to the higher rate of SAPs activity is clearly seen. Earlier, most mixes exhibit similar AE rates from the start of the monitoring, while at approximately 2 h the AE evolves to a lower rate. This initial period of high activity before 2 h coincides well with the measured settlement in cement, showing once again the sensitivity of the AE sensors to the micro-level processes [7].

**Figure 6:**
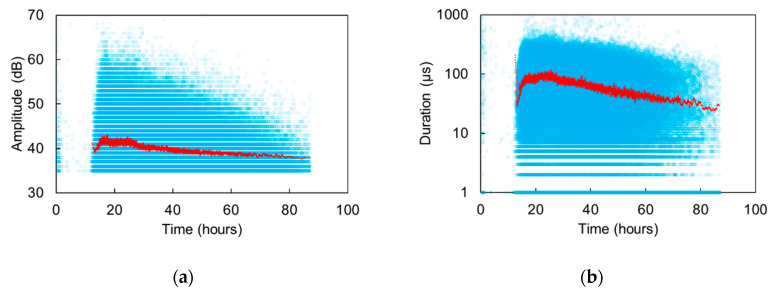
Amplitude (**a**) and duration (**b**) of AE waveforms versus time for a typical mortar specimen with SAPs. Each point stands for the amplitude of one AE signal and the red line stands for the moving average of 250 points.

Looking at the cumulative AE activity, a nearly constant rate is depicted for several hours during the activation of SAPs (i.e., at least between 12 and 30 h). However, more detail is offered by AE parameters like the amplitude and duration. In Figure 6a, it is clear that from the moment of the onset of the phenomenon (approximately 11 h as aforementioned), a rapid increase in the amplitudes is noted, reaching values of even 70 dB at 17–18 h. This level is maintained until approximately 26 h, also illustrated by the moving average red line of 250 points included in the graph when a gradual decrease starts to occur and continues until the end of monitoring at 85 h. Similar conclusions are provided in Figure 6b, where the AE duration is depicted. There, the average line starts at approximately 17 μs at 11 h, reaches a plateau of 100 μs until 26 h of curing and then gradually decreases to the initial level throughout the rest of the monitoring period. The results are in agreement with NMR data that show that these specific SAPs release water from final setting, at approximately 11 h after mixing, and most entrained water is released in between 22 h to 30 h, and then levelling down to slower pace when studied in sealed conditions [22]. This is also an indication that despite the inherently large scatter of AE data, quantitative information can still be drawn to accurately characterize microstructural processes and to determine the time frame for internal curing by the SAPs.

It is also noticed that different processes can be discriminated based on the AE characteristics. For example, the activity received during the period dominated by settlement (roughly first two hours), shows 50 to 400 times higher average energy (ranging between 500 and 10,000 attoJ) than the activity during the steady state of SAPs action (at approximately 25 h, ranging between 10 and 50 attoJ). In accordance, the typical duration of settlement AE signals (270–370 μs) is 3 to 4 times longer than SAPs activity signals (70–130 μs). Therefore, something that years ago seemed impossible (characterization of sources in fresh cement) and caused a lot of confusion to researchers, now starts to become substantiated and offers unique insights in the hidden processes within fresh cementitious media.

**Figure 7:**
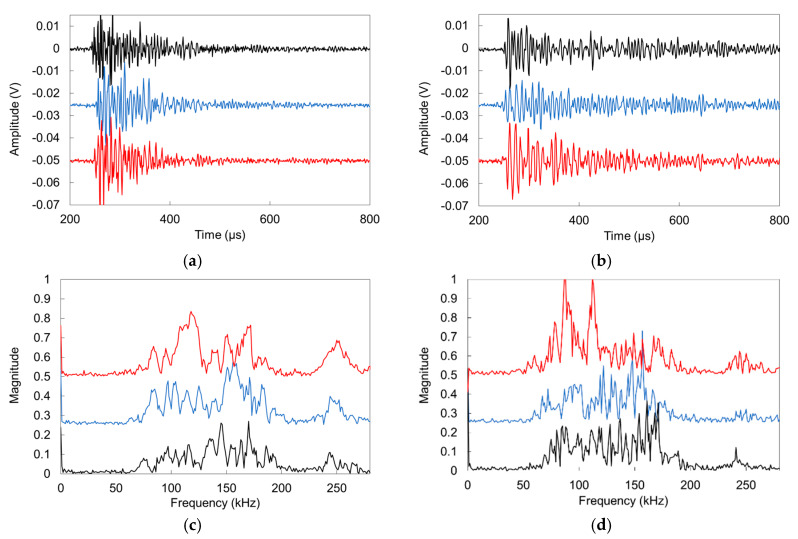
Typical AE waveforms: (**a**) during SAP activity (25 h after mixing) and (**b**) during settlement (<1 h after mixing). Typical FFT magnitudes are shown in (**c**) for SAP activity signals and in (**d**) for settlement signals (several signals are translated on the vertical axis for readability).

Selecting ‘representative’ AE waveforms is not straightforward due to the inherent experimental scatter of the parameters. However, it is always important to have a look at the raw data on which the analysis is based. Figure 7a,b show three AE signals from the period of intense SAP action (at 25 h), and the settlement (first 2 h) respectively. The waveform shapes do not fundamentally differ in shape, apart from the longer average duration of settlement signals in Figure 7b. Figure 7c,d show the corresponding FFT of the same waveforms. The main content is in any case in the band 50 to 200 kHz, which is expected reasonable due to the resonance of the sensors, while occasionally the magnitude of settlement signals Figure 7d reaches higher values than of SAPs action.

### 4.2. Ultrasonic Measurements on the Hardened Material

**Figure 8:**
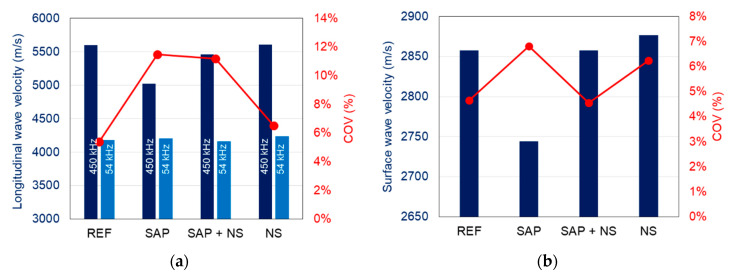
Elastic wave velocity and coefficient of variation of various mixes: (**a**) longitudinal waves and (**b**) surface or Rayleigh waves. Results of 450 kHz refer to the measurements with pico sensors after pencil lead excitation and 54 kHz refers to the experiments conducted with the commercial high- power ultrasonic device.

Figure 8a,b show the longitudinal and Rayleigh wave velocities respectively as measured by the pico sensors on the surface after pencil lead break. The results confirm the effect of the cavities created by the SAPs as the longitudinal wave velocity drops for the specific mix by more than 10%, while the Rayleigh wave velocity decreases by 4%. In addition, the beneficial effect of NS is also evident since the velocity of the mix with SAPs and NS is restored to the same level as the reference mix (above 5500 m/s). Mortar containing only NS exhibits even higher Rayleigh velocity values than the reference mix Figure 8b), due to the reinforcement by the nanomaterial. Considering the densities of all mixes, the Young’s moduli of the materials range between 46 GPa for SAPs mix and 55 to 57 GPa for the other mixes. A point that should be highlighted is the larger influence on the wave velocity when NS is added to SAP samples, compared to the addition to reference mixtures. This may be explained by the formation of products, caused by the pozzolanic nature of the nanosilica, within the macropores created by the emptying of the SAPs. Further research is however necessary to substantiate this assumption.

The influence of heterogeneity in the form of cavities is not only demonstrated by the lower velocity values but also by the experimental scatter they exhibit. The more heterogeneous the material, the more random it becomes, which is depicted in the coefficient of variation (COV) values, calculated as the standard deviation over the average and shown again in Figure 8a,b. Indicatively, while the COV for longitudinal waves of reference mortar is 5%, it increases to more than 11% for material with SAPs. Concerning the typical measurement error and taking into account the sampling rate of 10 MHz (time step 0.1 μs), this is calculated at an average of 0.98% for the longitudinal and at 0.56% for the Rayleigh wave velocities.

**Figure 9:**
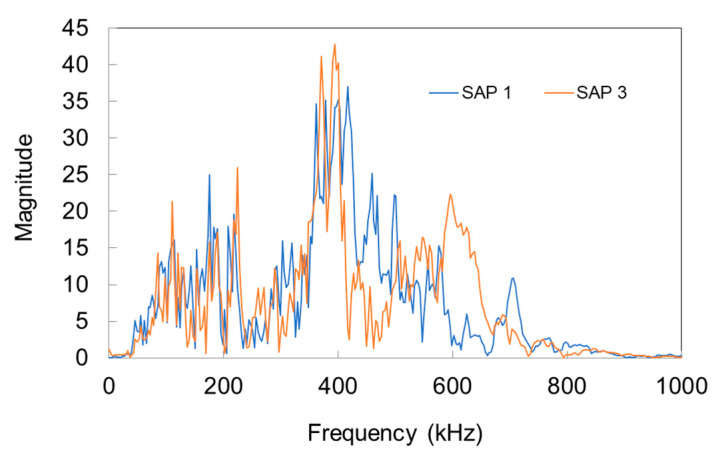
Typical FFT spectra from waveforms received on the surface of mortars with SAPs.

As aforementioned, apart from the surface measurements, ultrasonic tests took place through the longitudinal axis with a frequency of 54 kHz. The results are also seen in Figure 8a. The velocity values are lower than the higher frequency ones, something normal due to the well-known dispersion exhibited by cementitious media [33,45]. In addition, it is seen that lower frequencies and therefore, longer wavelengths do not help much to characterize between the various mixes in this scale, as the results are all within a range of 80 m/s (4160 m/s to 4240 m/s), without strong characterization power over the mixes. Indeed, 54 kHz results in wavelength λ of approximately 70–80 mm. Considering the dimensionless parameter α = πD/λ, where D is the inclusion diameter (SAP cavities have a maximum diameter size of 300 μm), and λ, the wavelength, it results in a value around 0.025, much lower than 1. This clearly indicates that the phenomena fall into the “long wavelength” regime [45], where limited interaction between the heterogeneity and the wavelength is expected. On the other hand, concerning the surface measurements mentioned above, Figure 9 shows typical spectra after pencil lead break excitation as received by the Pico sensors used for wave measurements on the surface. The main peaks come at approximately 400 kHz resulting in a representative Rayleigh wavelength of 7 mm. For this wavelength, the corresponding value of parameter α is 0.26, one order of magnitude higher than for the 54 kHz measurements. Therefore, although still lower than 1, the surface wave measurements after pencil lead excitation start to deviate from the long wavelength regime and the microstructure starts influencing more critically the results through scattering. In addition, there is substantial content even at higher frequencies up to 600 kHz, which would result in even smaller wavelengths, higher α values and stronger interaction.

**Figure 10:**
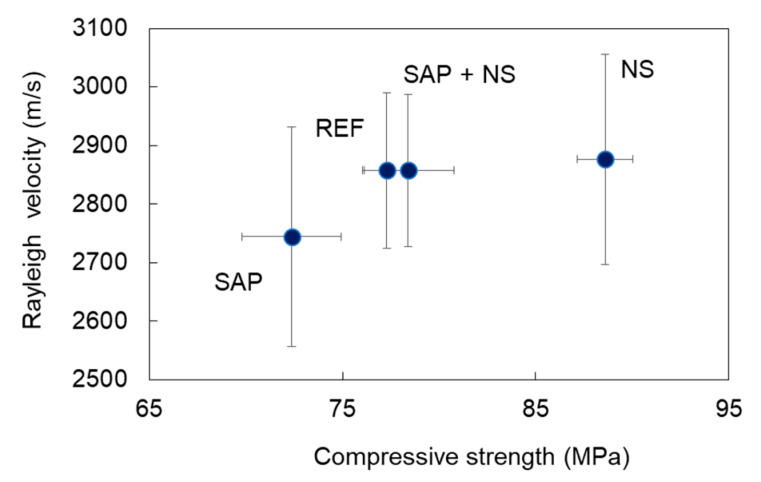
Correlation between average Rayleigh wave velocity and compressive strength of mortars.

One correlation that is also worth mentioning is the one between wave velocity and strength. While this is well known for cementitious materials, this is the first time that it is confirmed for this type of admixtures. Mortar with SAPs indeed exhibits simultaneously the lower strength and the lower Rayleigh wave velocity, while mortar with NS has the highest values exhibiting 22% higher strength and 5% higher velocity than the SAPs mixes Figure 10.

The above results demonstrate clearly that NDT based on elastic waves can be used to characterize advanced mixes as wave velocity correlates well with the expected microstructure and final mechanical properties, extending the knowledge from conventional materials to innovative cementitious mixes.

### 4.3. Drying-Wetting Cycles

**Figure 11:**
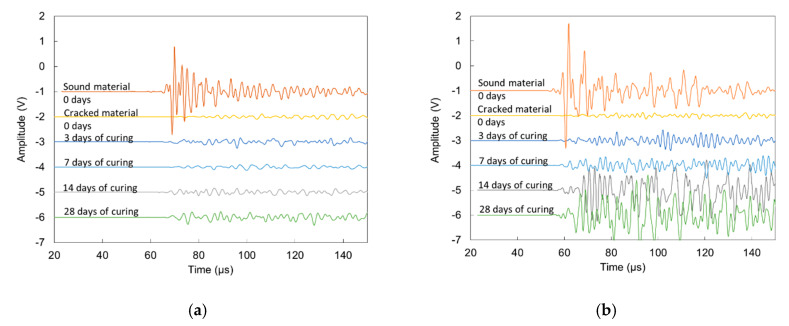
Waveforms received at successive stages of curing for (**a**) reference mortar sample and (**b**) SAP + NS mortar sample.

As aforementioned, healing cycles were conducted to check the capacity of crack closure and possible mechanical restoration. Initially, the specimens with rebars were loaded until no new cracks initiated. Upon reloading, the specimens were cured in wet-dry cycles, as explained in the experimental section. At specific ages, surface wave measurements were conducted with the two sensors placed at either side of a crack to check the effect of wet-dry curing on the signal transmission through possible sealing or healing. Specifically, the samples were studied six times (sound and cracked condition at 0 days and later at 3, 7, 14, 28 days during wet-dry cycles). The waveforms in Figure 11a correspond to the 2nd receiver on a reference sample. It is seen that after the crack occurrence, the waveform (2nd from top) loses much of its amplitude compared to the “sound” one (top waveform), while the Rayleigh cycle cannot be identified any longer. Throughout the wet-dry cycles, there seems to be an increase of the energy of the waveform, without however, being able to clearly detect the Rayleigh cycle similarly to before cracking. The increase can be due to the closure of the crack from late hydration products and calcium carbonate precipitation. This result is comparable to monitoring the healing capability on impacted plates with and without SAPs, by means of resonance analysis using a tap hammer [46]. Figure 11b shows a typical case for a SAP + NS specimen. The initial waveforms show the same tendency compared to the reference specimen, since after cracking, the transmission is seriously decreased, as seen by the reduction of amplitude. At later times however, the waveform starts to restore its content, signifying that more energy passes through the volume of the crack, while at 28 days, the Rayleigh peak becomes visible again, although not as clear as the one before cracking. This restoration of wave energy was noticed in most of the SAP + NS specimens while other mixes showed much weaker restoration. This is the first time that surface wave amplitude is used to monitor the crack closure effect of stimulated autogenous healing while in the past, it has been used for crack closure after epoxy repair [37].

**Figure 12:**
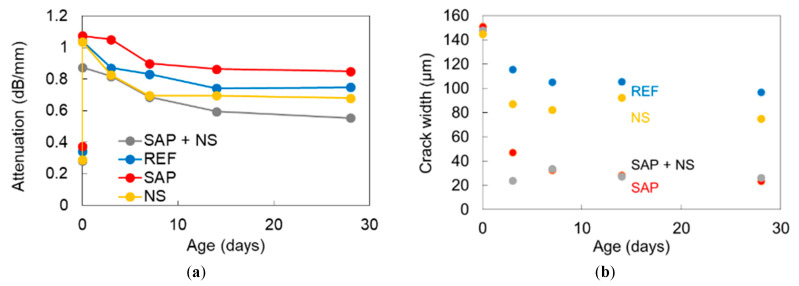
(**a**) Surface wave attenuation and (**b**) crack width for various mortar mixes vs. curing time in wet-dry cycles.

Considering all the monitored locations (four different cracks for each mix during 28 days of wet-dry cycles), an average value for the attenuation coefficient can be calculated. It is measured by the ratio of the maximum amplitude of the waveform of the 2^nd^ receiver over the maximum amplitude of the 1^st^ “reference” receiver (close to excitation, receiving the signal before passing through the crack), divided over the sensor to sensor distance of 30 mm and expressed in dB. The low values below 0.4 dB/mm at 0 days, as shown in Figure 12a, correspond to the attenuation of the sound media before cracking. Just after cracking, the attenuation strongly increased, to values around 1 dB/mm showing the influence of the discontinuity on the wave path. As the wet-dry cycles are performed, the attenuation of all mixes shows a decreasing trend, evident of the fact that cracks are closing due to further hydration products that are formed between the crack sides and the deposition of calcium carbonate. In addition to the general decreasing trend, it is obvious that the attenuation of SAP + NS mortars exhibit much lower values than the other mixes signifying much better transmission conditions through the volume of the crack. 

Wave attenuation can be discussed in relation to the microscopy results on the same mixes that were cracked in bending and followed the same wet-dry cycles, as explained earlier. Figure 12b shows the average crack width for the four considered mixtures: reference, SAP, NS and SAP + NS. 

**Figure 13:**
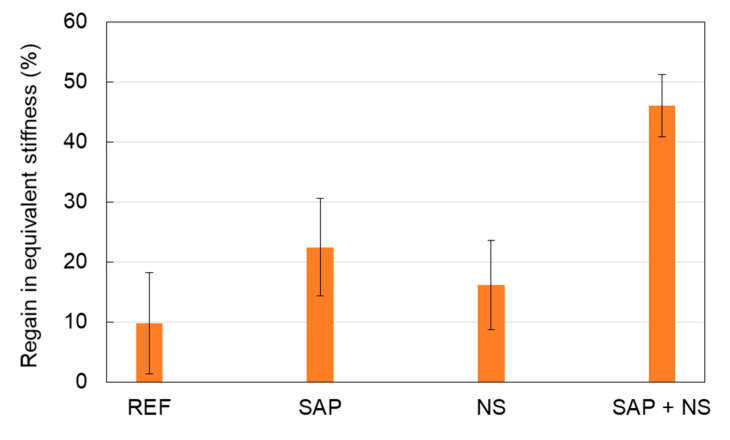
Regain in equivalent stiffness (slope of load-displacement curves) of all mixture series.

The initial crack is very similar as this is imposed by the metal frame where the specimens are fit into after the mechanical cracking, thus the value of 150 μm is the starting point to check the healing or sealing potential of the different compositions. From the second measurement at 3 days, the average readings of the cracks were reduced, even for the mixtures without SAPs showing a value below 120 μm and 90 µm for reference and NS samples respectively. For SAP and SAP + NS compositions, the average crack width exhibited a much stronger reduction being close to 40 μm. Wet-dry cycle curing until 28 days has a small additional effect especially for the SAP specimens, which exhibit an average final crack width of 24 μm while for SAP + NS the final value was 34 μm. Therefore, microscopy results confirm that the addition of SAPs contributes to the closure of cracks, either as standalone admixture or in combination with NS. In general, the trend of decreasing attenuation is in agreement with the closing trend of cracks, initially exhibiting stronger rate and later being saturated. However, there is one point that needs to be highlighted. While SAP and SAP + NS mixes exhibit similar crack closure at 28 days in Figure 12b the attenuation shows much lower values for SAP + NS mixes than SAPs alone. The reason behind these differences is likely to be caused by the variation on the initial crack widths that exists for the specimens with rebars, studied for the attenuation profile. An average value, considering all initial crack width measurements, was equal to 89 ± 33 μm for SAP + NS samples, 139 ± 64 μm and 139 ± 48 μm for reference and NS, respectively, while for SAP specimens a mean crack width of 202 ± 102 μm was found. The significantly larger average crack width in SAP samples can lead to a limited total healing, as the total amount of healing products necessary to fully close the cracks is higher compared to the SAP + NS series. This trend, indicated by the lower attenuation of SAP + NS, was tested by mechanical loading, where the same specimens used for surface wave measurements were reloaded in tension after 28 days of wet-dry cycles. The average regain in equivalent stiffness of all mixtures is shown in Figure 13. The equivalent stiffness, measured by the slope of the load-displacement curve, during reloading is compared to the one of the loading stage. A regain of only 10% was seen for the reference samples, while for the SAP specimens this regain was increased up to 22%. This means that, even though the crack widths in the SAP specimens were on average wider compared to the reference material, the healing ratio is still higher for SAP inclusion. This is due to the promotion of further hydration by the SAPs by nearly 40%, as confirmed earlier by NMR measurements [17] and visualized by means of X-ray tomography [47]. When comparing the SAP and SAP + NS samples, better healing conditions are given for the latter series. This is due to the stronger initial cementitious matrix, resulting in smaller crack widths compared to cracks in the reference and NS specimens. The included SAPs also improved further hydration in this case. Moreover, the addition of NS had a positive influence on the healing capacity when compared to the reference series. In this case, the average crack openings were comparable. A possible explanation could be the formation of other healing products, caused by the pozzolanic nature of the nanomaterial, promoting the healing capability. The latter phenomenon would confirm the restoration of stiffness for SAP + NS, being much stronger than for other mixes, like the restoration of signal transmission implied through the decrease of attenuation. This is subject for further research.

For completeness, it is mentioned that the assessment after cracking is based on the amplitude and not on the wave velocity for two basic reasons. The first is that the wave amplitude (or inversely attenuation) is much more sensitive to the cracks and heterogeneity in general, as well known from the literature [33,34,35,36] and also shown in this study. In the examples of Figure 11a,b above, the peak amplitude drops by 95% after the crack (compare “sound material” and “cracked material” waveforms). At the same time, judging from the onset of these waveforms, the velocity drops by about 50% (from approximately 5500 m/s to approximately 2800 m/s in both cases). Therefore, amplitude shows much stronger sensitivity and characterization capacity for the same cracking than the velocity. Furthermore, an important point is that after the development of the crack, the reliability of picking the onset of the waveform is compromised due to the low amplitude of the opening cycles of the waveform. These peaks are quite low and similar to the noise level, reducing the reliability of a specific pick for the onset. This of course does not hold for the attenuation, which is measured by the peak amplitude of the waveform and can be clearly depicted in all cases.

## 5. Conclusions

This paper studied the use of elastic wave NDT as a promising method to monitor the various processes occurring in cementitious materials and to characterize their inner microstructure. The mixtures under study contained different additives, being SAPs to mitigate autogenous shrinkage and NS to counteract the reduction in strength caused by SAP inclusion. The effects of these components on the hydration process, the final microstructure and the self-healing efficiency were measured by wave methods and a comparison with the results of more common experimental procedures was made.

Acoustic emission monitoring of reference and SAP mortars revealed the action of the SAPs during hydration. A steep increase in received AE hits was noticed between approximately 11 h and 40 h of curing in case of SAP samples, whereas this was not seen for the reference mortars. The increase in hits therefore can be linked to the release of water by the SAPs as the desiccation of the mortar and internal curing initiate. Moreover, by analysis of the received waveforms, the action of the SAPs could be distinguished from the settlement, occurring within the first hours of curing.

Secondly, using ultrasonic measurements performed by pencil lead break tests, differences in microstructure between the four mixtures were exposed by variations in longitudinal and surface wave velocities measured. The creation of cavities after water release by the SAPs lowered both wave velocities and increased the scatter on the results, while the inclusion of NS increased the wave velocity due to the reinforcement of the matrix.

Finally, it was seen that the addition of SAPs and NS improved the self-healing capacity of the mortar specimens. Tensile tests were performed to obtain multiple cracking and after 28 days of healing in wet-dry cycles, the tensile reloading showed that a partial regain in stiffness could be obtained for the SAP + NS mixtures. During these wet-dry cycles, ultrasonic tests were conducted next to specific crack openings to receive information on waves travelling across the crack opening. By examination of the attenuation, decreasing over time as curing in wet-dry cycles was performed, the increased healing capability of the SAP + NS mixtures compared to other mixtures was confirmed.

The improved healing capacity of SAP + NS mixtures, determined by means of wave measurements and regain in mechanical stiffness and confirmed by the results of water permeability tests and visual crack closure [21] is an interesting feature of the newly obtained cementitious material. Together with the mitigation of autogenous shrinkage through the inclusion of SAPs, without having a negative influence on the compressive strength, the combination of SAPs and nanosilica shows to be a promising addition to cementitious mixtures, meeting the continuously increasing requirements regarding the performance of construction materials.

## Figures and Tables

**Table 1:** Ratios of mixture components with respect to the binder content.

	Cement	Water	Sand	Superplasticizer	SAP	Dry NS
Reference	1	0.35	2	0.004		
0.2% SAP	1	0.402	2	0.004	0.002	
2% NS	0.98	0.35	2	0.076		0.02
0.2% SAP + 2% NS	0.98	0.402	2	0.076	0.002	0.02

**Table 2:** Density (g/m³) and compressive strength (MPa) of the four mixtures under study, measured at 28 days of curing.

	Density (g/cm³)	Compressive Strength (MPa)
Reference	2.16 ± 0.01	77.29 ± 1.17
0.2% SAP	2.17 ± 0.05	72.36 ± 2.55
2% NS	2.20 ± 0.01	88.60 ± 1.44
0.2% SAP + 2% NS	2.16 ± 0.02	78.39 ± 2.37
